# Editorial: Mental health characteristics of migrant and local populations in the prevention and management of mental health disorders

**DOI:** 10.3389/fpsyt.2023.1348239

**Published:** 2023-12-14

**Authors:** Ovais Wadoo, Javed Latoo, Yousaf Iqbal, Muhammad Naeem, Majid Alabdulla

**Affiliations:** ^1^Department of Psychiatry, Hamad Medical Corporation, Doha, Qatar; ^2^College of Medicine, Qatar University, Doha, Qatar; ^3^Lancashire and South Cumbria NHS Foundation Trust, Preston, United Kingdom

**Keywords:** mental health, migrants, refugee, local population, cultural competence, access to healthcare services

This Research Topic endeavors to contribute to the understanding of mental health characteristics among migrant and local populations. The overarching goal is to shed light on the multifaceted nature of migration, exploring its impact on mental wellbeing through assessments and evidence-based interventions. This initiative aims to expand the existing corpus of literature in the field and four key studies form the foundation of this exploration.

Migration, a multifaceted and dynamic phenomenon influenced by various factors such as economic conditions, political situations, environmental changes, and individual aspirations, encompasses diverse categories of migrants driven by distinct motives. Economic migrants seek improved financial prospects, while refugees flee persecution, and climate migrants are displaced due to environmental changes (1). Forced migrants, including internally displaced persons, are compelled to leave their homes within their country, and mixed migration involves a combination of motives (2). As of 2020, ~281 million people globally had migrated, with 82.4 million being forcibly displaced individuals, including 26.4 million refugees (3).

The mental health of migrants is intricately connected to socioeconomic factors, with poverty, unemployment, discrimination, and other determinants influencing an individual's wellbeing. Housing instability, neighborhood conditions, and access to healthcare services also play roles in mental health outcomes (4). Social support, derived from community and networks, is crucial for mental wellbeing, while the societal stigma surrounding mental health and cultural norms impact help-seeking behavior. Early life experiences, workplace factors, and access to resources further contribute to mental health disparities (5). The research by Pham et al. delves into the association between social support and the development of depressive symptoms among storm-affected Vietnamese Americans, recommending prioritization of social support resources in the aftermath of hurricanes.

Refugees, forced to leave their home countries due to persecution or conflict, often experience trauma and face challenges adapting to new cultures, languages, and social norms (6). Homesickness is a profound and complex emotional experience prevalent among refugee populations, stemming from forced displacement and the subsequent loss of home, community, and loved ones. Refugees often grapple with cultural dislocation, navigating unfamiliar environments with different languages and customs, exacerbating feelings of alienation. The uncertainty surrounding their future, be it a return home or permanent resettlement, further intensifies homesickness, creating a sense of limbo. Social isolation, difficulty forming new relationships, and nostalgia for the familiarity of their homeland contribute to a deep emotional yearning. Additionally, refugees face identity struggles as they try to integrate into a new society while holding onto their cultural roots (7). The study by Rosner et al. explored homesickness in refugees, linking it to mental health symptoms and migration-related factors.

Special migrant groups, including pregnant individuals, the elderly, those with disabilities, those with physical co-morbidities, and children, face unique challenges. Pregnant migrants may experience acculturation stress and concerns about healthcare access during pregnancy. The elderly among migrants may face social isolation and health-related anxieties, compounded by language barriers. Migrants with disabilities confront challenges related to accessibility and social inclusion, impacting mental wellbeing. Those with physical co-morbidities require integrated healthcare addressing both physical and mental health aspects, with cultural competence playing a pivotal role.

Migration can also significantly impact those left behind. The migration of children, while providing economic opportunities for the children, significantly impacts the wellbeing of parents left behind. Parents experience a mix of emotions, including loneliness, anxiety, and depression. The absence of emotional support, financial dependence on remittances, and communication challenges contribute to stress. Worrying about the safety and wellbeing of migrated children adds to parental stress, while societal expectations and stigma may affect the social standing of parents within their communities. Lu et al. explore the effect of children's internal migration on the subjective wellbeing of parents left behind, emphasizing the negative impact and reduction of intergenerational spiritual support.

Mental illness presentations vary significantly among diverse cultures due to distinct cultural norms, beliefs, and expressions of psychological distress. Cultures influenced by collectivism may prioritize familial and societal harmony, potentially leading to the underreporting of individual mental health concerns. Some cultures use spiritual or religious frameworks to understand and cope with mental illness, while others may have specific idioms of distress not easily classified within diagnostic categories. Understanding these cultural nuances is crucial for effective mental health interventions. Barriers to mental health services for migrants include language differences, cultural disparities, and lack of awareness (8). Both migrants and local populations may underreport mental health issues due to stigma. Many individuals in both groups demonstrate resilience, drawing on personal and community strengths to cope with stressors. Comprehensive strategies addressing both individual and systemic factors are essential, including improved access to culturally competent mental health services, social inclusion, stigma reduction, and consideration of economic and social determinants. Culturally tailored interventions, trauma-informed care, and collaboration with multidisciplinary teams are crucial for addressing the unique needs of migrants, especially those who may have experienced trauma during migration. Additionally, addressing barriers to service access, providing affordable options, and offering flexible service models, including telehealth, promote inclusivity. Education and awareness campaigns are vital for reducing stigma and promoting mental health literacy. Training for service providers should include understanding acculturation, discrimination, and social determinants of mental health (9). Prevention, early intervention, and policy advocacy support mental health equity, ensuring equal access to quality services for both migrant and local populations. Advocacy for policies that support mental health equity ensures equal access to quality services for both migrant and local populations, ultimately fostering the overall wellbeing of diverse communities. Lastly, the work by Spanhel et al. investigates the feasibility, acceptance, and preliminary effectiveness of a culturally adapted digital sleep intervention for refugees, suggesting scalable approaches to making mental healthcare accessible.

In conclusion, effective mental health service delivery for migrants and the local population requires a culturally sensitive, inclusive, and multidisciplinary approach. Mental health professionals must be culturally competent, understanding and respecting diverse backgrounds. The diverse experiences within each group necessitate a nuanced understanding of mental health presentations across different cultures. Fostering the wellbeing of diverse communities, regardless of migration status, demands a comprehensive, inclusive, and culturally sensitive approach to mental health service delivery.

## Author contributions

OW: Conceptualization, Writing—original draft, Writing—review & editing. JL: Writing—review & editing. YI: Writing—review & editing. MN: Writing—review & editing. MA: Writing—review & editing.
